# Rapid and High-Efficiency Laser-Alloying Formation of ZnMgO Nanocrystals

**DOI:** 10.1038/srep28131

**Published:** 2016-06-21

**Authors:** Peisheng Liu, Hao Wang, Jun Chen, Xiaoming Li, Haibo Zeng

**Affiliations:** 1 Institute of Optoelectronics & Nanomaterials, Jiangsu Key Laboratory of Advanced Micro & Nano Materials and Technology, College of Material Science and Engineering, Nanjing University of Science and Technology, Nanjing 210094, China; 2 Jiangsu Key Laboratory of ASCI Design, College of Electronics and Information, Nantong University, Nantong 226019, China; 3 College of Science, Nantong University, Nantong 226019, China

## Abstract

Applications of ZnMgO nanocrystals (NCs), especially in photoelectric detectors, have significant limitations because of the unresolved phase separation in the synthesis process. Here, we propose a rapid and highly efficient ZnMgO NC alloying method based on pulsed laser ablation in liquid. The limit value of homogeneous magnesium (Mg) is pushed from 37% to 62%, and the optical band gap is increased to 3.7 eV with high doping efficiency (>100%). Further investigations on the lattice geometry of ZnMgO NCs indicate that all ZnMgO NCs are hexagonal wurtzite structures, and the (002) and (100) peaks shift to higher diffraction angles with the increase in Mg doping content. The calculated results of the lattice constants a and c slightly decrease based on Bragg’s law and lattice geometry equations. Furthermore, the relationship between annealing temperature and the limit value of homogeneous Mg is examined, and the results reveal that the latter decreases with the former because of the phase separation of MgO. A probable mechanism of zinc magnesium alloy is introduced to expound on the details of the laser-alloying process.

Semiconductor oxide materials based on energy-gap engineering have garnered widespread interest in many aspects, for instance, in catalysts, sensors, electronic devices, detectors and solar cells, among others^1^,^2^,^3^,^4^,^5^,^6^,^7^,^8^,^9^,^10^,^11^. Among these semiconductor oxide materials, zinc oxide (ZnO) has attracted considerable attention because of its wide band gap of 3.37 eV and high exciton binding energy of 60 meV, which is much larger than that of GaN (25 meV) and ZnSe (22 meV)^12^. To utilize the optical and electrical properties of ZnO sufficiently, an excellent method is to dope proper transition elements, such as Co, Mn, Fe, Ni, etc^13^,^14^,^15^. Among which, Mg is an appropriate element because Mg-doped ZnO has a theoretically wide band gap scope from 3.37 eV to 7.8 eV. Its slight lattice mismatch is attributed to the large band gap (7.8 eV) of MgO and the close proximity of ionic radii between Mg^2+^ (0.57 Å) and Zn^2+^ (0.60 Å)^16^,^17^,^18^.

Recently, many studies have been conducted on zinc-magnesium (Zn-Mg) alloys by manifold methods such as metal organic vapor phase deposition and molecular beam epitaxy, among others^19^,^20^. However, the products obtained by the two methods have serious phase separation at a low Mg-doped concentration (about 37% at^18^.) ascribed to the limit solubility of Mg in the ZnO lattice. Moreover, the conditions of high temperature, high pressure, and protected gas are required in the synthesis process, which restricts their popular applications. In this study, our group develops a general and facile method based on laser ablation for Zn-Mg alloy with highly doped Mg content and high doping efficiency. To the best of our knowledge, only a few reports have been conducted on Zn-Mg alloys by laser ablating Zn-Mg alloy targets with different atomic percentages in liquid. Compared with other methods, laser ablation in liquid (LAL) has many advantages: it requires a simple operation, does not require high temperature, high pressure, and protected gas, produces an extreme non-equilibrium condition, and has a highly active plasmon plume in the rapid synthesis process, among others. Moreover, it can improve the Mg solubility in ZnMgO NCs. Therefore, LAL may be beneficial in promoting Mg dopants diffusing into the base compound.

Here, we report on the rapid and high-efficiency alloy of ZnMgO NCs by pulsed laser ablating Zn-Mg alloy targets with different atomic percentages. Through this laser-alloying method, the Mg^2+^ ions can be introduced into the ZnO lattice without phase separation with concentration as high as 62%. The optical band gap is pushed to 3.7 eV with high doping efficiency (>100%). The investigation of the lattice geometry indicates that the lattice constants a and c of ZnMgO NCs slightly decrease with the increase in Mg content. The relationship between the annealing temperature and the limit value of homogeneous Mg is also investigated. The results reveal that the latter decreases with the former because of phase separation of MgO. According to the analysis above, a probable Zn-Mg alloy mechanism is proposed to expound on the details of the laser-alloying process.

## Results

### Structural properties of ZnMgO NCs

**Figure 1:**
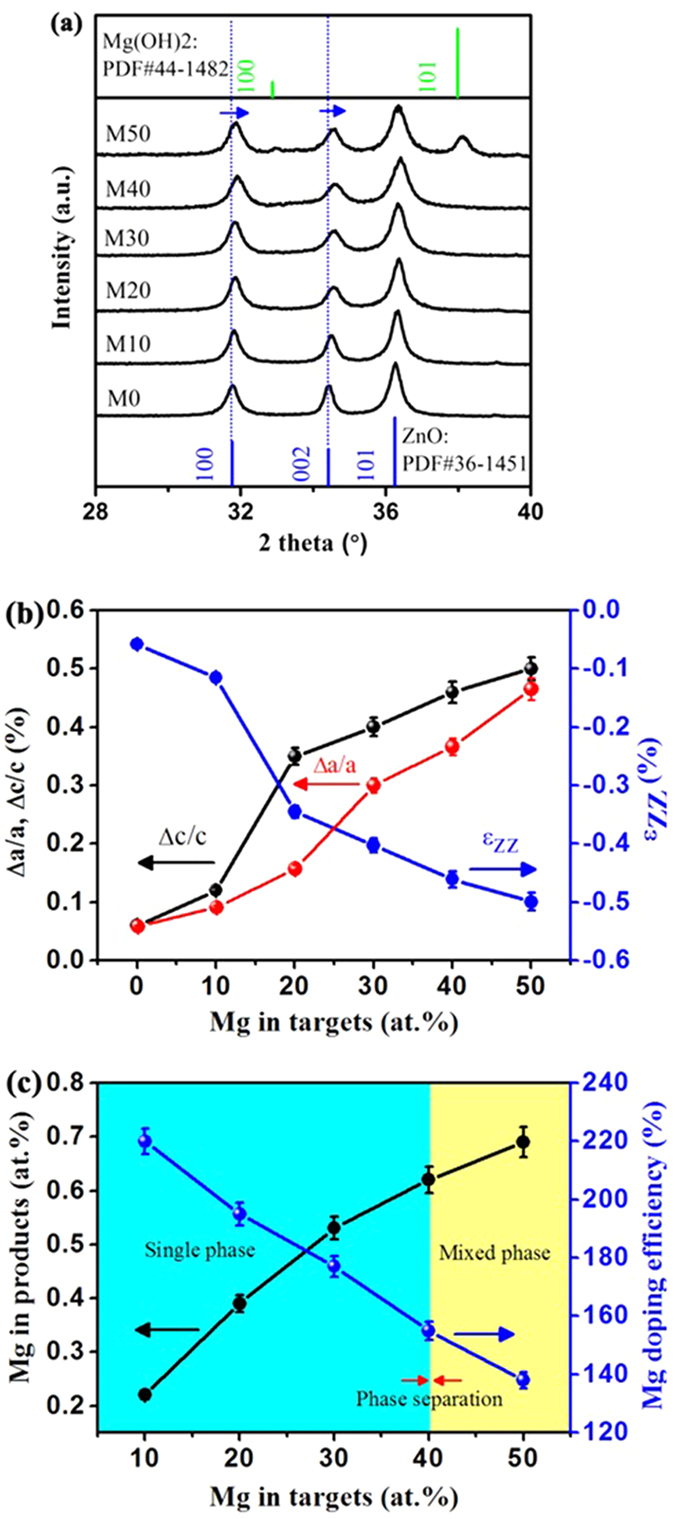
(**a**) XRD patterns of ZnMgO NCs with different atomic percentage of Mg in the alloy targets. The blue and green lines at the bottom and top represent the standard diffraction peaks of hexagonal ZnO and Mg(OH)_2_, respectively. (**b**) Mg content dependences of the lattice constants ∆a/a, ∆c/c and lattice strain of ZnMgO NCs calculated from the results of XRD. (**c**) Evolution of Mg content in ZnMgO NCs and doping efficiency as functions of Mg content in alloy targets.

**Figure 2:**
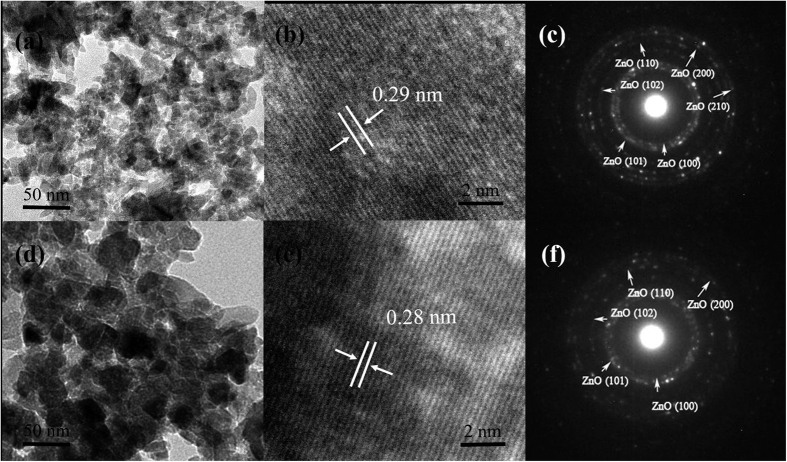
(**a,d**) TEM images of the nanostructures from the representative ZnMgO NCs of M10 and M50, respectively. (**b,c**) and (**e,f**) are the corresponding high-resolution TEM (HRTEM) images and SAED patterns, respectively.

By pulsed laser ablating Zn-Mg alloy targets with different atomic percentages in deionized water, Mg ions can be introduced into the ZnO lattice as confirmed by X-ray diffraction (XRD) patterns (Fig. 1a) and lattice evolutions (Fig. 1b). Figure 1a presents the XRD patterns of ZnMgO NCs prepared with an atomic percentage of Mg (*X*_*n*_) in the 0–50% range. With an increase in *X*_*n*_ from 0% to 40%, the wurtzite characteristic peaks of ZnO (PDF#36-1451) can be observed but not the (101) diffraction peak of Zn crystal at 0% (Fig. S1), thus indicating the formation of phase-pure ZnMgO NCs. As *X*_*n*_ increases to 50%, small peaks corresponding to the hexagonal Mg(OH)_2_ (101) plane (PDF#44-1482) appear along with the wurtzite features. The lattice structure of ZnMgO NCs with *X*_*n*_ from 0% to 50% is greatly affected by dopants as revealed in Fig. 1b. First, the broadening of diffraction peaks implies the slight degradation of crystallinity attributed to dopant-induced lattice deformation and crystal size decrease as shown in Fig. 2. Furthermore, with the increase in *X*_*n*_, the (100) and (002) diffraction peaks shift from 31.76° to 31.91° and from 34.42° to 34.61°, respectively. These lattice contractions in the a-axis and c-axis, which are distinct from those in the one-pot thermolysis of metal slats in our previous report^21^, are further confirmed by lattice geometry equations and Bragg’s law^22^,^23^,^24^,^25^ as follows:


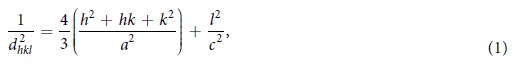



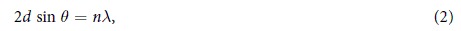


The first-order approximation, *n* = 1, is obtained. Thus, for the (100) and (002) orientations, the lattice constants a and c are calculated by


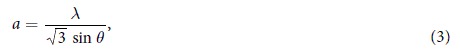



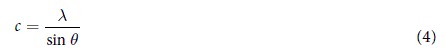


The lattice constants a and c both decrease from 3.253 Å to 3.238 Å and from 5.210 Å to 5.184 Å with the increase in *X*_*n*_ (Tables S1 and S2), respectively. More importantly, the dependence of lattice deformation on *X*_*n*_ is approximately linear in the single-phase range as shown in Fig. 1b. Clearly, the absolute value of lattice strain increases with the increasing *X*_*n*_ in Fig. 1b. According to previous reports^26^,^27^, the values of lattice strain in ZnO can be calculated by the following equation:


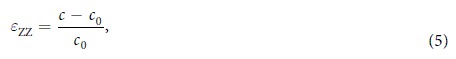


here, *ε*_ZZ_ is the lattice strain, *c*_0_ and *c* are the lattice constants in the raw and Mg-doped samples, respectively (Table S2). The variation of lattice strain in ZnO is attributed to the presence of impurities, defects, and lattice distortions in the crystal. These lattice strain evolutions also indicate that large amounts of Mg^2+^ ions have been introduced into the lattice at the Zn^2+^ ion-substituted sites.

To examine the factual alloying degree, Mg content in ZnMgO NCs is measured by inductively coupled plasma-atomic emission spectrometry (ICP-AES) as shown in Fig. 1c. The evolution of Mg content in ZnMgO NCs (*X*) increases with the increase in *X*_*n*_. However, phase separation occurs when*X*_*n*_ > 40% (marked yellow in Fig. 1c), and mixed phases that include wurtzite ZnO and hexagonal Mg(OH)_2_ are observed. The simple wurtzite ZnO phase is marked green in Fig. 1c. The doping efficiencies (*X*/*X*_*n*_) of Mg are all beyond 100% with Mg having high solubility of up to 62% (Table S3) when *X*_*n*_ < 40%. This high doping efficiency may originate from the major loss of Zn^2+^ in the laser-alloying process, which leads to the sharply decrease of Zn content in ZnMgO NCs. Because of the difference in the physico-chemical properties between Zn^2+^ and Mg^2+^, the loss of Zn^2+^ in the laser-alloying process easily occurs at the extreme non-equilibrium condition induced by LAL, compared with Mg^2+^. This major loss of Zn^2+^ results in the high doping efficiency, which even exceeds 100%. However, doping efficiency appears to decrease with the increasing *X*_*n*_, which is attributed to the low solubility of Mg in the hexagonal ZnO lattice originating from the difference in crystal structures.

The nanostructures from the representative products of *X*_*n*_ = 10% and 50% were studied according to the transmission electron microscopy (TEM) images and elected area electronic diffraction (SAED) patterns, as shown in Fig. 2. Figure 2a,d indicate that ZnMgO NCs are spindle or flake nanostructures similar to the other obtained ZnMgO NCs of *X*_*n*_ = 20%, 30%, and 40% (Fig. S2) with good crystallinity. The slight difference in crystal plane spacing is 0.29 nm (*X*_*n*_ = 10%) and 0.28 nm (*X*_*n*_ = 50%) as clearly revealed in Fig. 2b,e, respectively. Interestingly, no obvious diffraction circles of Mg(OH)_2_ are observed according to the SAED patterns, although the apparent diffraction peaks appear in the XRD patterns (Fig. 1a). This phenomenon is attributed to the faintest content of Mg(OH)_2_.

### Optical properties of ZnMgO NCs

**Figure 3:**
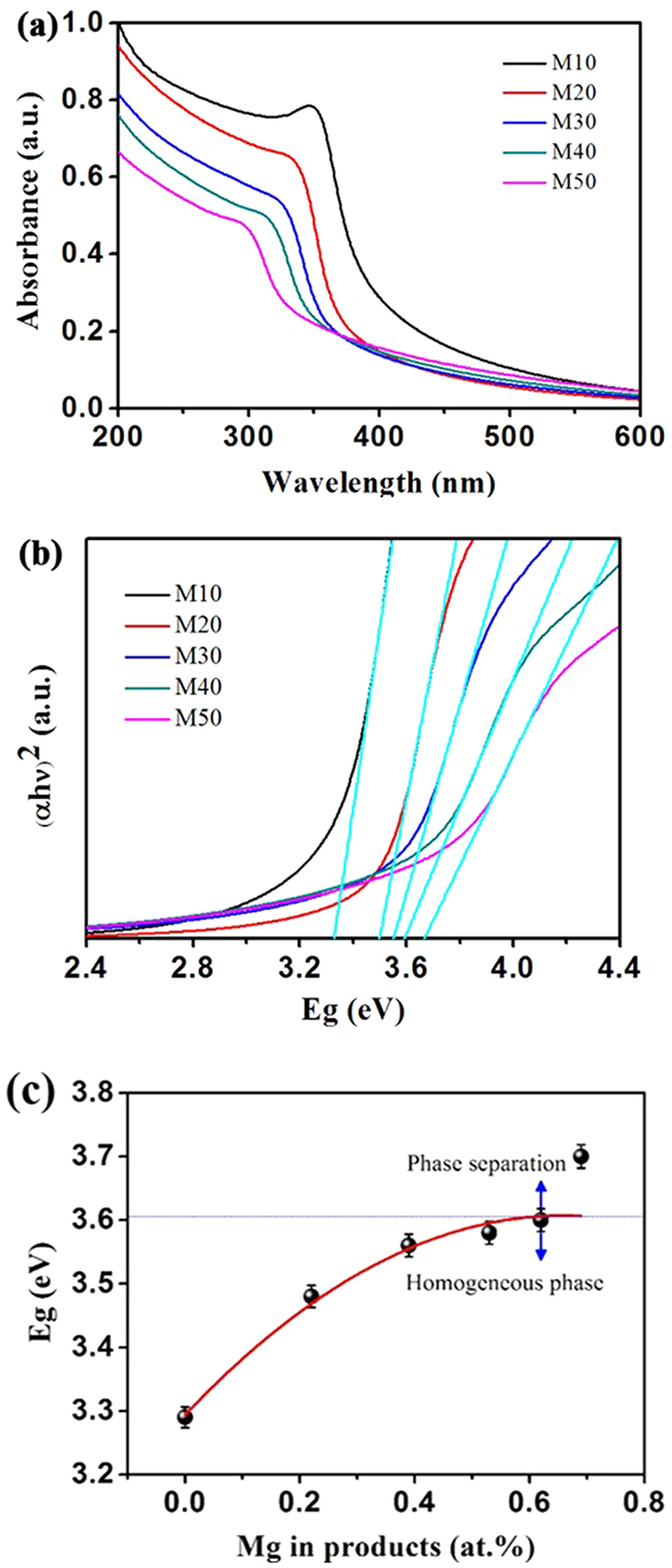
(**a**) UV-vis absorbance spectrum of ZnMgO NCs M10, M20, M30, M40, M50, respectively. (**b**) The plot of (*αhν*)^2^ vs. photon energy, where *α* is the absorption coefficient, respectively. The extrapolation of the linear region of the plot gives the value of the optical band gap. (**c**) Variation of the band gap as a function of Mg content in ZnMgO NCs. The fitting curve is marked as red in (**c**).

To further investigate the influence of Mg dopants on optical properties, ultraviolet-visible (UV-vis) absorbance spectrum of ZnMgO NCs are presented in Fig. 3. The band edge absorption of ZnMgO NCs has an apparent blue shift with *X*_*n*_ from 10% to 50% (Fig. 3a). The Mg^2+^ ions have also been introduced into the ZnO lattice at Zn^2+^ ion-substituted sites. The un-doped ZnO has an n-type doping profile. As the structure is doped with Mg, the exitonic transition energy blue-shifts to the high energy value that corresponds to the short-wavelength region. Figure 3b presents the evolutions of the optical band gaps calculated on the basis of Tauc’s relation:


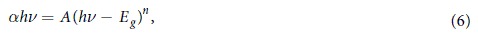


where *n* = 1/2 for the direct band gap semiconductor materials^17^,^28^. The optical band gaps of ZnMgO NCs increase from 3.48 eV to 3.70 eV with the increase in *X*_*n*_ from 10% to 50% (Table S4). Furthermore, doped Mg in the ZnO lattice leads to the decreases in lattice constants a and c with an increase in the band gap energy structure. Therefore, the blue shift of the band edge absorption of ZnMgO NCs can be explained by the evolutions in lattice constants^1^,^29^. In the Zn-Mg alloying process, Mg^2+^ ions substitute the Zn^2+^ ionic sites without phase separation up to *X* = 62%, which is much greater than the limit value of homogeneous Mg of 37%^18^, because of the extreme non-equilibrium condition and the highly active plasmon plume in the rapid synthesis induced by LAL (Fig. 3c). Nonetheless, phase separation also occurs as *X* of up to 69%, and it is attributed to the limit solubility of Mg in the ZnO lattice and the slight divergence of ionic radii between Mg^2+^ and Zn^2+^. The red fitting curve (Fig. 3c) further confirms that the variation of the optical band gaps is nonlinear with the increase in*X*, consistent with other reports^17^,^18^,^21^.

**Figure 4:**
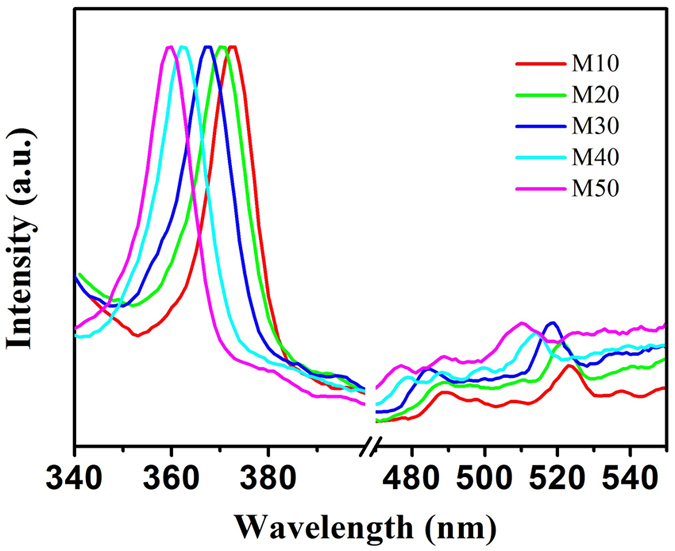
Normalized PL spectrum of ZnMgO NCs M10, M20, M30, M40, M50, respectively. Excitation wavelength: 320 nm.

Moreover, we also investigate the normalized photoluminescence (PL) spectrum of ZnMgO NCs using 320 nm excitation wavelength, as presented in Fig. 4. Obviously, the absorption peaks of ZnMgO NCs blue-shift from approximately 375 nm to 354 nm with the increase of Mg content in ZnMgO NCs, consistent with that in their band edge absorption. This blue shift in the absorption peaks is originated from Mg dopant, which also induces the blue shift in blue and green fluorescence because of the widening defect states in ZnMgO NCs.

### Effect of annealing temperature

**Figure 5:**
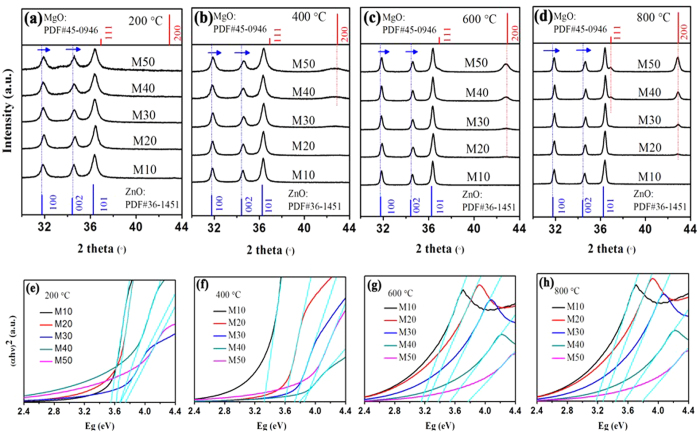
(**a–d**) XRD patterns of ZnMgO NCs annealed at 200 °C, 400 °C, 600 °C, 800 °C under N_2_ atmosphere, respectively. The blue and red lines at the bottom and top represent the standard diffraction peaks of wurtzite ZnO and cubic MgO, respectively. (**e–h**) are the corresponding plots of (*αhν*)^2^ vs. photon energy, respectively.

As ZnO has added intrinsic defects induced by Mg dopants in ZnO, annealing experiments were conducted to demonstrate the evolutions of structures and optical properties. The XRD patterns of ZnMgO NCs after annealing and the corresponding evolutions of the optical band gaps are presented in Fig. 5. All ZnMgO NCs have excellent crystallinities. That is, the defects decrease with the (100) and (002) plane diffraction peaks shifting to high diffraction angles as mentioned above. Along with the increased annealing temperature from 400 °C to 800 °C, cubic phase MgO appears because of phase separation of MgO formed by the Mg migration induced by annealing when *X*_*n*_ = 40%, 30%, and 20%, respectively. However, no obvious diffraction peaks of cubic MgO and hexagonal Mg(OH)_2_ appear with the annealing temperature of 200 °C because of the discrepancy of crystallinity among ZnO, MgO, and Mg(OH)_2_ (Fig. 5a). Moreover, all the diffraction peaks become narrower and sharper, thus further confirming that the intrinsic and added defects in ZnMgO NCs decrease sharply and Mg^2+^ ions have been introduced into the ZnO lattice.

We also examine the corresponding variation of the optical band gaps, as indicated in Fig. 5e,f and Table 1. With the increasing annealing temperature from 200 °C to 800 °C, the optical band gap of each annealing temperature has a shift trend toward the high energy band region. However, a slight difference occurs in the increasing value, consistent with the blue shift in the UV-vis absorbance spectra (Fig. S3). The increasing optical band gap is generally attributed to the evolutions in lattice constants caused by Mg dopant. The substitution of Zn^2+^ by Mg^2+^ leads to a decrease in *c*/*a* ratio because of the divergences in the electronegativity and ionic radius between Zn^2+^ and Mg^2+ ^1,20. Moreover, Mg dopant also lifts the bottom of ZnO conduction band, which is attributed to occurrence of s- and p-Mg originated band states. During the substitution of Zn^2+^ by Mg^2+^ process, the local structural rearrangement appears in ZnO lattice with the oxygen (O) electronic distribution shifting to Mg. As Mg doping into the ZnO lattice, the 4s-Zn electron states, which determine the bottom of ZnO conduction band, can shift to the higher energy band region. And the valence band position of ZnO is generally determined by the 2p-O electron states, which remains virtually steady^29^. Therefore, it appears a widening of the optical band gaps in. ZnMgO NCs with an increase in Mg-doped concentration. Moreover, annealing conditions (e.g., annealing temperature, gas atmosphere, etc.) can promote crystallinity and reduce the defect concentrations, both of which greatly affect the crystal structures and optical properties of ZnMgO NCs.

**Figure 6:**
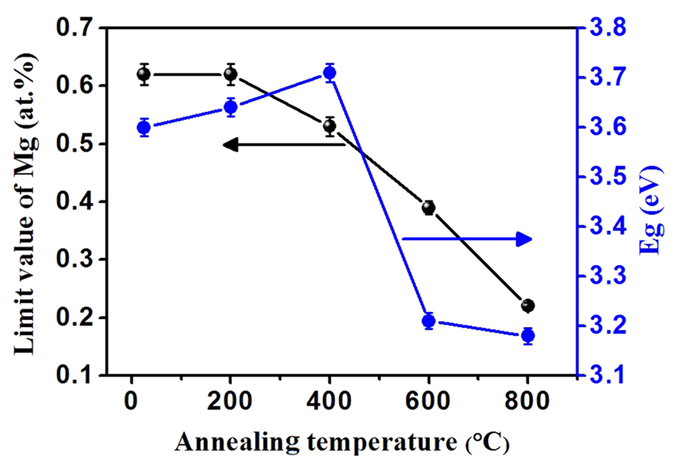
Evolution of limit values of homogeneous Mg in ZnMgO NCs and the corresponding band gap with the increasing annealing temperature, respectively.

However, the evolution of the limit value of homogeneous Mg and the corresponding optical band gaps with the increasing annealing temperature are slightly inconsistent, as shown in Fig. 6. With the increase in annealing temperature up to 800 °C, the decreased limit value of homogeneous Mg is attributed to the phase separation of MgO generated by annealing. The band gap initially increases and then reduces sharply because of the competitive effect between the defects and the Mg^2+^ (Fig. 6). At a low Mg-doped concentration, the intrinsic and added defects in ZnMgO NCs affect the energy band structure, and the resultant Mg^2+^ has a predominant role of substituting the defects with the increasing Mg-doped content. Initially, the defects in energy band structure decrease with the increasing annealing temperature from room temperature to 400 °C with good crystallinity and widening optical band gap of ZnMgO NCs. Subsequently, along with the sustainably increasing annealing temperature of up to 800 °C, the optical band gap sharply decreases because of the separation of large quantities of Mg from ZnMgO NCs induced by annealing. Compared with the primary effect of the defects, the separation of large quantities of Mg has a greater effect on the energy band structure after 400 °C. Therefore, we conclude that the defects and Mg dopants in ZnO have predominant effects on the energy band structure at low and high Mg-doped concentrations, respectively.

### Formation mechanism of ZnMgO NCs

**Figure 7:**
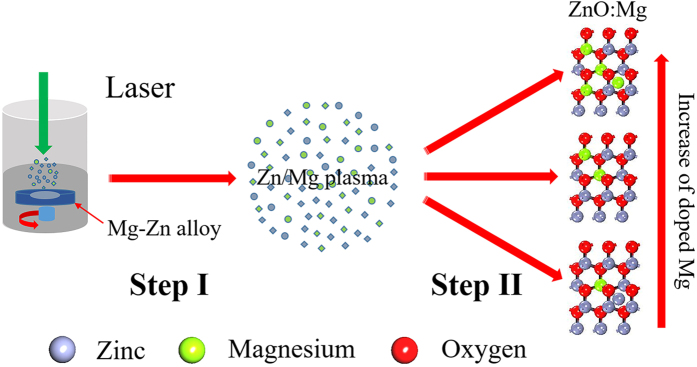
Illustration of Zn-Mg alloy. Step I: Production of high-temperature and high-density Zn-Mg plasma above the alloy target rapidly after one pulse shot. Step II: Subsequent ultrasonic and adiabatic expansion of the plasma leads to the cooling of the Zn-Mg plume region and hence to formation of the different constituent ZnMgO NCs with extinguishment of the plasma.

In the past decades, alloy materials of semiconductor ZnO have been extensively applied in many aspects because of their superior optical and electric properties. To better understand the process of Zn-Mg alloy, the alloy formation mechanism is described in Fig. 7. To the best of our knowledge, only a few reports have been conducted on the mechanism of Zn-Mg alloy by LAL. According to our sufficient experimental data and analysis, the probable mechanism of Zn-Mg alloy can be depicted in two steps as illustrated in Fig. 7. (I) The high-temperature and high-density Zn-Mg plasma (without solvent) is produced at the solid-liquid interface quickly after the irradiation of the pulsed laser on Zn-Mg alloy targets. (II) The subsequent ultrasonic and adiabatic expansion of the high-temperature and high-density Zn-Mg plasma leads to the rapid cooling of the Zn-Mg plume region and to the formation of Zn-Mg alloy clusters along with the extinguishment of the formed Zn-Mg plasma. In our case, the interval between two successive pulses is 0.1 s (10 H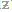), which is much longer than the life of the plasma plume (approximately 1000 ns)^30^. Therefore, the following laser pulse has no interaction with the former plasma plume.

As the Zn-Mg alloy clusters encounter the surrounding deionized water, they become oxidized by oxygen dissolved in deionized water or decomposed by water induced by LAL. However, ZnMgO NCs with low Mg content also contain small Zn crystal because of the incomplete oxidization of Zn originating from the protection of the ZnO layer encapsulated on the Zn surface. Thus, a bit of interstitial Zn can be found in the alloy structure as clearly indicated in Fig. 7. With increasing *X*_*n*_ in the Zn-Mg alloy targets, the Mg^2+^ ions substitute more Zn^2+^ ionic sites in the ZnO lattice without phase separation, with Mg concentration as high as*X* = 62% and the extinguishment of Zn crystal. When *X*_*n*_ increases to 50%, more Mg^2+^ ions are introduced into the ZnO lattice, and the excess Mg, which has no contribution to the widening of the optical band gap, enters into the clearance of the alloy structure to form interstitial Mg or magnesium hydroxide with phase separation (Fig. 1a).

Although we do not avoid the problem of phase separation originating from the limit solubility of Mg in the Zn-Mg alloy process, we have improved the limit solubility of homogenous Mg in the ZnO lattice up to 62% without phase separation and with high doping efficiency (>100%) because of the extreme non-equilibrium condition and highly active plasmon plume in the rapid synthesis by LAL. This facile alloy method is considered beneficial and effective for alloys of other materials.

## Discussion (Conclusion)

In summary, a simple and highly efficient approach was proposed to achieve the alloy of ZnMgO NCs using pulsed laser ablating Zn-Mg alloy targets. The Mg^2+^ ions could be introduced into the ZnO lattice without phase separation with concentration as high as 62%. The optical band gap was increased up to 3.7 eV with high doping efficiency (>100%). Further investigations on the lattice geometry of ZnMgO NCs indicated that all ZnMgO NCs were hexagonal wurtzite structures with diffraction peaks shifting to higher diffraction angles with the increase in Mg-doped content. The calculated results of the lattice constants a and c slightly decreased based on Bragg’s law and lattice geometry equations. The effects of annealing temperature demonstrated that the defects and the Mg dopants in ZnO predominantly affected the energy band structure at low and high Mg-doped concentrations, respectively. According to the experimental results and analysis above, a probable mechanism of Zn-Mg alloy was presented to elaborate on the details of the laser-alloying process. The simple and rapid laser-alloying method may be beneficial to other material alloys.

## Methods

### Nanocrystal synthesis

The representative experiment^31^,^32^ was conducted through pulsed laser ablation of Zn-Mg alloy targets (purity 99.9%, atomic percentage of Zn-Mg alloy from 10:90 to 50:50, diameter 50 mm, thickness 5 mm) and pure Zn target (purity 99.9%) in deionized water. The targets were fixed on the bracket in a glass vessel filled with 25 mL solution that was continuously stirred to disperse the smoke-like colloids above the metal target. The plate was placed 4 mm from the solution surface in the solution and then was ablated by the first harmonic (1064 nm) of an Nd: YAG laser operated at 10 Hz with a pulse width of 10 ns. A Scientech power meter monitored the output of the 1064 nm laser with power density of 6.37 J/(cm^2^∙pulse). The laser beam was focused on the alloy targets with a spot size of about 2 mm in diameter using a lens with a focal length of 200 mm. Laser ablation time lasted for 2 h, and the colloidal solutions turned to milk white from initially colorless. After ablation, the solutions were centrifuged at 9000 rpm for 3 min, and the as-prepared powder-products were draught-dried in atmosphere at room temperature for 12 h. Lastly, the draught-dried products were placed in the rapid annealing furnace and annealed at 200~800 °C for 2 h in N_2_ atmosphere with a flux of 100 sccm per minute. The temperature increased at the rate of 20 °C/min and then decreased to room temperature in N_2_ atmosphere.

### Material characterization

For TEM and HRTEM examination, the as-prepared powder products were ultrasonically re-dispersed in ethanol, before they were dropped on the copper grids coated with thin carbon film and evaporated in air at room temperature. TEM and HRTEM observations were conducted on a JEOL 2010 TEM, operating at an accelerating voltage of 200 kV. XRD patterns were recorded on a multipurpose XRD system D8 Advance from Bruker with a Cu Kα radiation (λ = 1.5406 Å). The optical absorption spectra of the obtained colloidal solutions were recorded immediately after laser ablation, by a Cary 5E UV-vis-IR spectrometer. Finally, the as-prepared colloidal solutions were ultrasonically dispersed for 30 min and then diluted by 5% hydrochloric acid. Subsequently, an 8 ml diluted solution was drawn out to conduct an ICP-AES test on the Shimadzu ICPE-9000 emission spectrometer.

## Additional Information

**How to cite this article**: Liu, P. *et al*. Rapid and High-Efficiency Laser-Alloying Formation of ZnMgO Nanocrystals. *Sci. Rep.* **6**, 28131; doi: 10.1038/srep28131 (2016).

## Supplementary Material

Supplementary Information

## Figures and Tables

**Table 1:** Evolutions of the optical band gaps of ZnMgO NCs unannealed and annealed at different temperature.

Samples	Band gap (eV)				
	Unannealed	200 °C	400 °C	600 °C	800 °C
M10	3.48	3.52	3.32	3.15	3.18
M20	3.56	3.61	3.60	3.21	3.27
M30	3.58	3.64	3.71	3.38	3.42
M40	3.60	3.64	3.79	3.51	3.52
M50	3.70	3.72	3.82	3.78	3.80
