# Supplementary material for: Rapid and High-Efficiency Laser-Alloying Formation of ZnMgO Nanocrystals
Source: Sci Rep. 2016 Jun 21;6:28131. doi: 10.1038/srep28131 (PMC4914852; doi:10.1038/srep28131)
Supplement: Supplementary Information [file srep28131-s1.doc]

*Supplementary Information*

**Rapid and High-Efficiency Laser-Alloying Formation of ZnMgO Nanocrystals**

*Peisheng Liu, ^1,2^ Hao Wang,^1,2,3^ Jun Chen,^1^ Xiaoming Li,^1^ Haibo Zeng^1^,**

*^1^Institute of Optoelectronics & Nanomaterials, Jiangsu Key Laboratory of Advanced Micro & Nano Materials and Technology, College of Material Science and Engineering, Nanjing University of Science and Technology, Nanjing 210094, China,*

*^2^Jiangsu Key Laboratory of ASCI Design, College of Electronics and Information Engineering, Nantong University, Nantong 226019, China,*

*^3^College of Science, Nantong University, Nantong 226019, China*

*Corresponding Author: [zeng.haibo@njust.edu.cn](mailto:zeng.haibo@njust.edu.cnm)

**
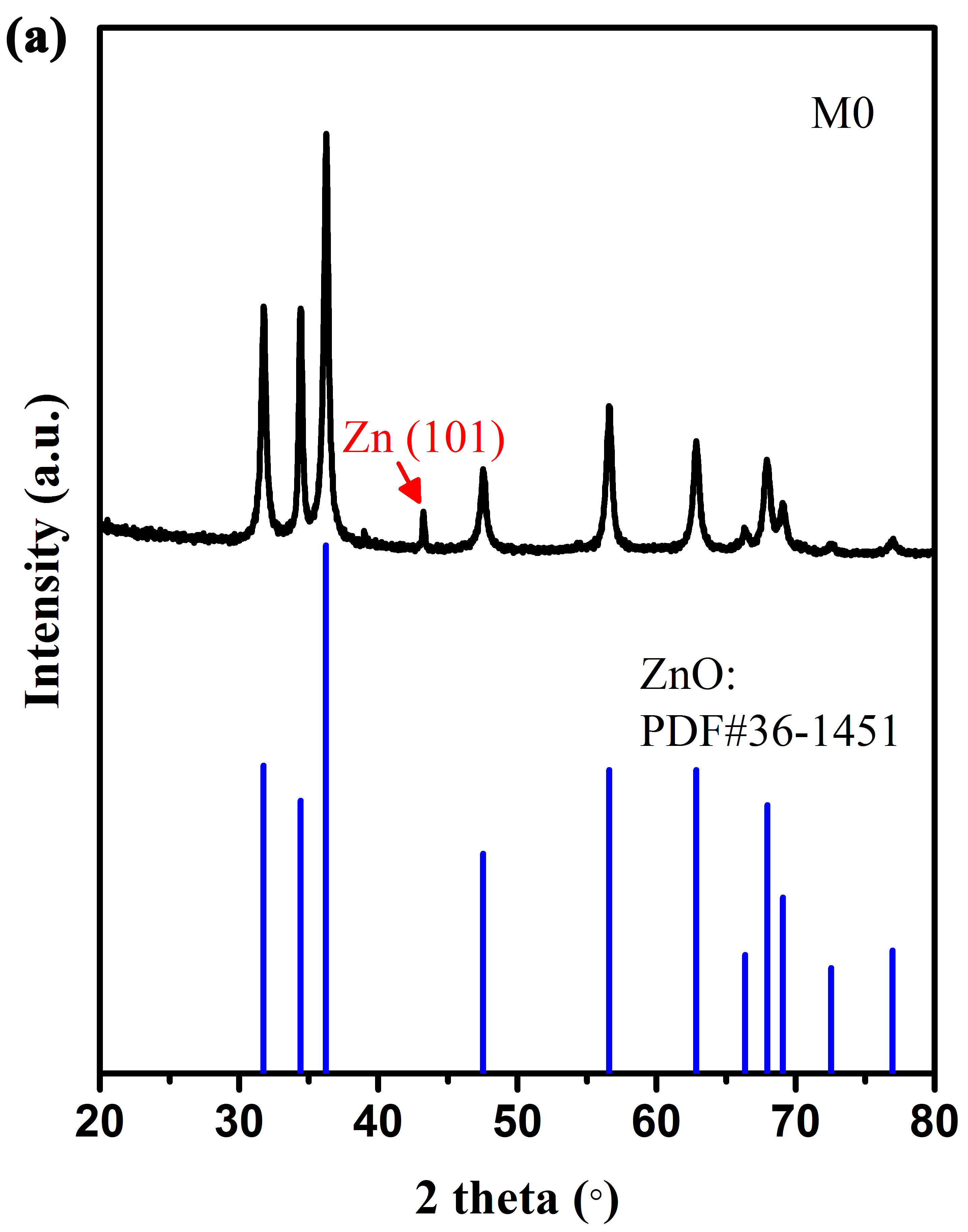
**

**
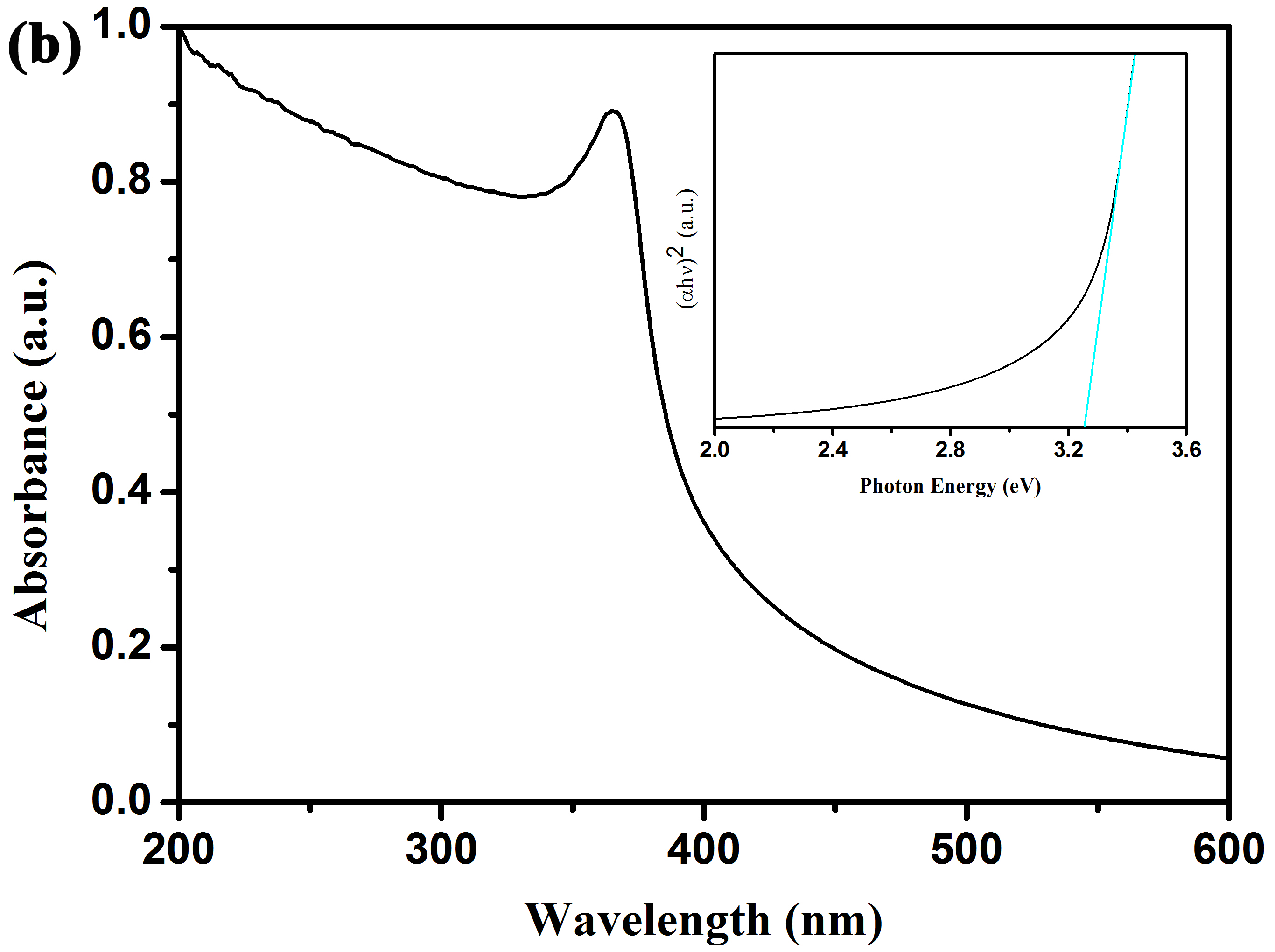
**

Figure S1. (a) XRD pattern of ZnO NCs by laser ablating pure zinc target in deionized water. The blue lines at bottom represent the standard diffraction peaks of the wurtzite ZnO (PDF#36-1451). Clearly, the (101) diffraction peak of Zn crystal appears in the ZnO NCs because of the incomplete oxidation of Zn in the synthesis process. (b) UV-vis absorbance spectrum of ZnO NCs (M0). Obviously, it has an absorption peak at approximate 370 nm, which corresponds to the intrinsic absorption of ZnO. The inset is the plot of $(\alpha h\nu)^{2}$ vs. photon energy, where $\alpha$ is the absorption coefficient. An extrapolation of the linear region of a plot of $(\alpha h\nu)^{2}$ vs. $h\nu$ presents the value of the optical band gap$E_{g}$. In this case, it is estimated about 3.29 eV, which is proximate to that in the pure ZnO (3.37 eV).

**
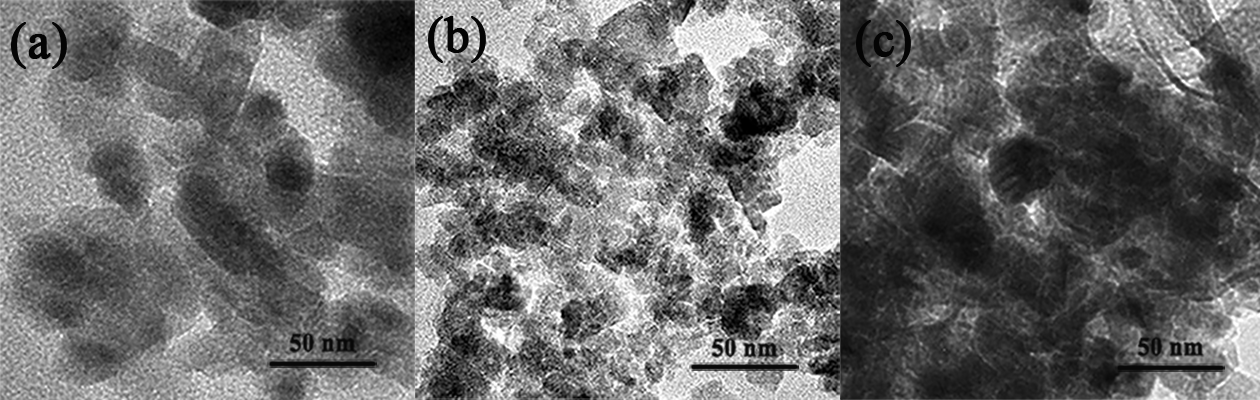
**

Figure S2. (a-c) TEM images of ZnMgO NCs of M20, M30, M40, and they presents the morphologies of spindle or flaky with the faintest aggregation because of the poor dispersion in deionized water. Moreover, they also possess a wide size rang around from 12 nm to 38 nm, and the influence of quantum size effect on the optical properties can be neglected essentially.

**
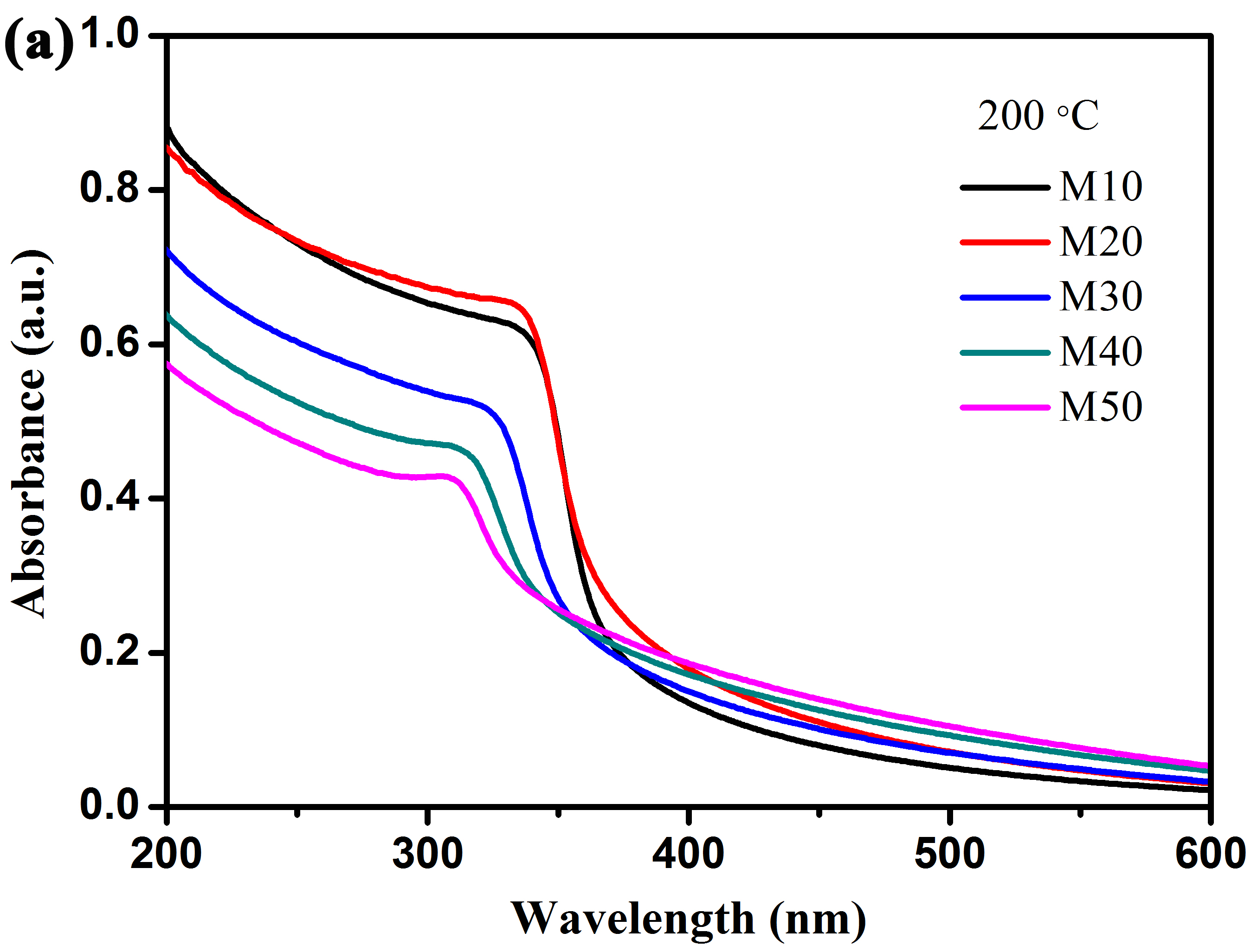
** **
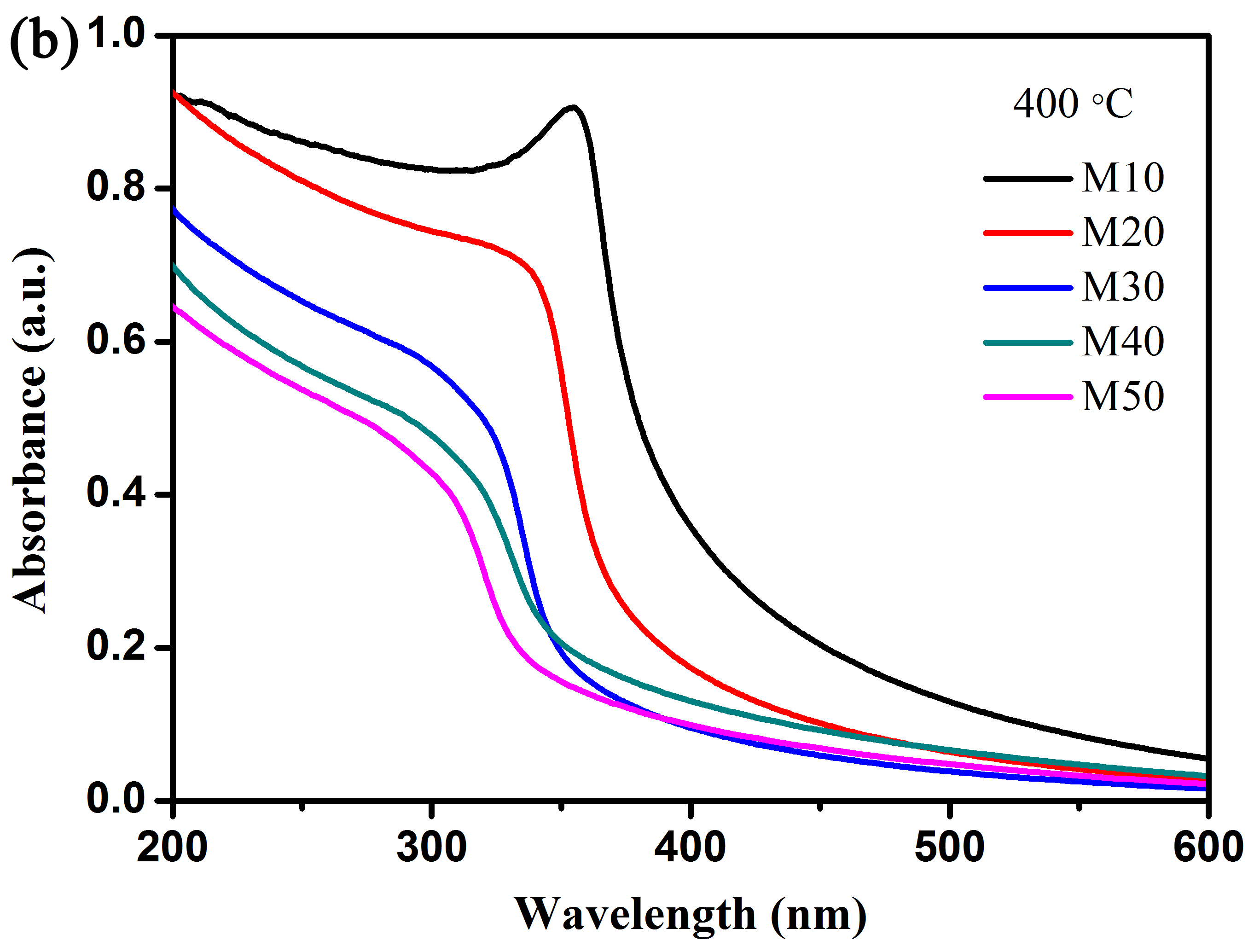
**

**
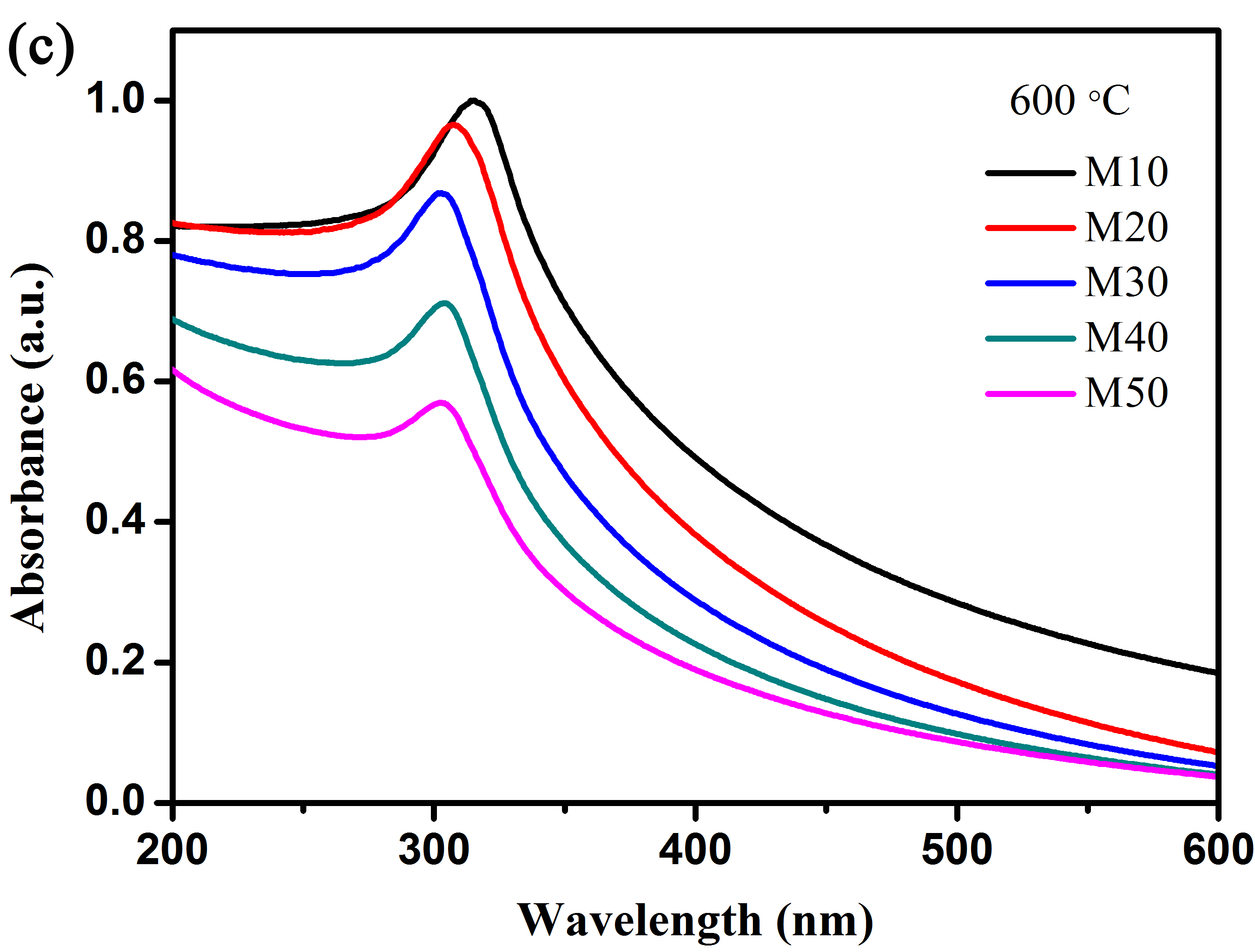
** **
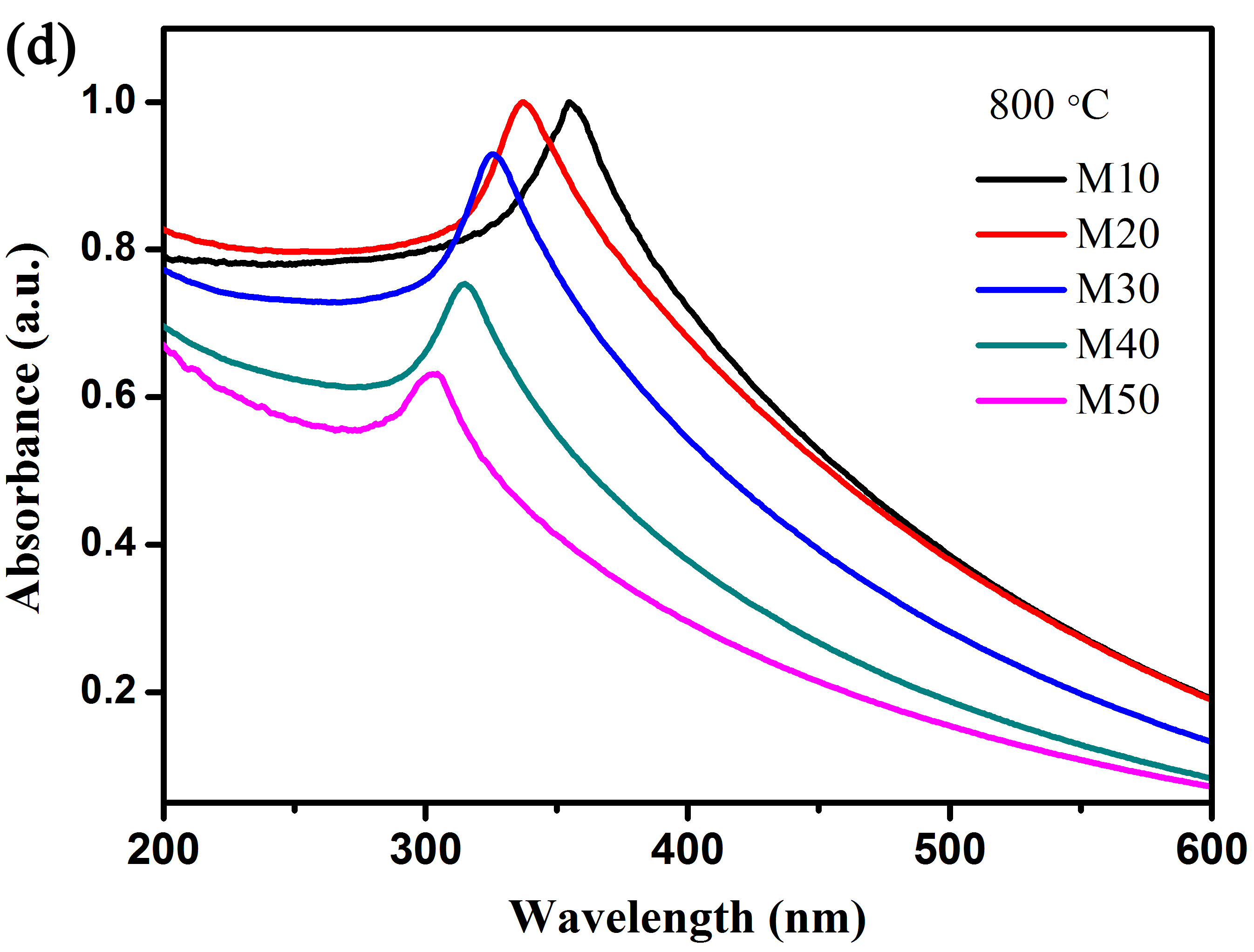
**

Figure S3. (a-d) UV-vis absorbance spectra of ZnMgO NCs annealed at 200 ºC, 400 ºC, 600 ºC, 800 ºC under N_2_ atmosphere, respectively. There exists an apparent blue-shift in each annealing temperature because of the decrease in the defects and phase separation of MgO in ZnMgO NCs originating from the effects of annealing. At a low annealing temperature, the defects primarily affect the structure and optical properties of ZnMgO NCs. On the contrary, the Mg dopant plays a principal role. Therefore, the evolutions of the optical band gap are attributed to the competitive results between the defects and Mg dopants.

| Samples | (100) / θ | (002) / θ | a / Å | c / Å |
| --- | --- | --- | --- | --- |
| M0 | 15.8830 | 17.2131 | 3.250 | 5.207 |
| M10 | 15.9035 | 17.2336 | 3.249 | 5.204 |
| M20 | 15.9137 | 17.2745 | 3.247 | 5.192 |
| M30 | 15.9342 | 17.2847 | 3.243 | 5.189 |
| M40 | 15.9444 | 17.2950 | 3.241 | 5.186 |
| M50 | 15.9546 | 17.3052 | 3.238 | 5.184 |

Table S1. Evolutions of diffraction angles of (100) and (002) and the corresponding lattice constants a, c of ZnMgO NCs.

| Samples | ∆a/a (%) | ∆c/c (%) | ɛ_zz_ / (%) |
| --- | --- | --- | --- |
| M0 | 0.06 | 0.06 | -0.058 |
| M10 | 0.09 | 0.12 | -0.115 |
| M20 | 0.15 | 0.35 | -0.345 |
| M30 | 0.28 | 0.40 | -0.403 |
| M40 | 0.34 | 0.46 | -0.461 |
| M50 | 0.43 | 0.50 | -0.499 |

Table S2. Rates of the change of the lattice constants a, c and variations of lattice strain $\varepsilon_{zz}$of ZnMgO NCs.

| Samples | Mg content (ppm) | Zn content (ppm) | Mg content (molar ratio ) | Zn content (molar ratio ) | Doping efficiency (%) |
| --- | --- | --- | --- | --- | --- |
| M10 | 1.027 | 9.611 | 0.22 | 0.78 | 220 |
| M20 | 2.701 | 10.99 | 0.39 | 0.61 | 195 |
| M30 | 3.709 | 8.820 | 0.53 | 0.47 | 177 |
| M40 | 6.642 | 11.41 | 0.62 | 0.38 | 155 |
| M50 | 9.418 | 11.36 | 0.69 | 0.31 | 138 |

Table S3. Data of Mg content obtained by the ICP-AES with high doping efficiency ($X/X_{n}$) and the corresponding molar ratios in ZnMgO NCs.

| Samples | M0 | M10 | M20 | M30 | M40 | M50 |
| --- | --- | --- | --- | --- | --- | --- |
| Band gap (eV) | 3.29 | 3.48 | 3.56 | 3.58 | 3.60 | 3.70 |

Table S4. Data of the optical band gaps of ZnMgO NCs by laser ablating different atomic percentage Zn-Mg alloy targets in deionized water.
